# Diabetes and disordered eating behaviours in a community-based sample of Australian adolescents

**DOI:** 10.1186/s40337-020-0282-y

**Published:** 2020-02-28

**Authors:** Kirrilly M. Pursey, Phillipa Hay, Kay Bussey, Nora Trompeter, Alexandra Lonergan, Kathleen M. Pike, Jonathon Mond, Deborah Mitchison

**Affiliations:** 1 Faculty of Health and Medicine, University of Newcastle, Callaghan, 2308 NSW Australia; 2 Translational Health Research Institute, School of Medicine, Western Sydney University, Sydney, NSW Australia; 3 Campbelltown and Camden Hospitals, Campbelltown, NSW Australia; 4 Department of Psychology and Centre for Emotional Health, Macquarie University, Sydney, NSW Australia; 5 Departments of Psychiatry and Epidemiology, Columbia University Irving Medical Center, New York, NY USA; 6 Centre for Rural Health, University of Tasmania, Launceston, Australia; 7 School of Medicine, Western Sydney University, Campbelltown, Australia; 8 Department of Psychology, Macquarie University, Sydney, NSW Australia

**Keywords:** Disordered eating behaviours, Diabetes, Eating disorders, Adolescence

## Abstract

**Background:**

People with diabetes have been shown to be at risk for disordered eating compared to their non-diabetic peers. However, the majority of studies have been conducted in relatively small samples drawn from clinical diabetes settings or registries. Community-based samples are required to better understand disordered eating behaviours in this population. In a large community-based population sample of Australian adolescents, this study aimed to (1) investigate disordered eating behaviours in adolescents reporting a diagnosis of diabetes compared to their non-diabetic peers and (2) test associations between disordered eating behaviours and insulin restriction.

**Methods:**

Secondary school students (*n* = 4854; mean (SD) age 14.4 (1.6) years; 47% boys) completed an online survey, including self-reported presence of diabetes, demographics, weight status, substance use, insulin restriction and disordered eating behaviours. Clinically meaningful cut-offs for disordered eating behaviours were generated for analysis.

**Results:**

Disordered eating behaviours, specifically self-induced vomiting (diabetes 19.2%, no diabetes 3.3%; *p* <  0.001), laxative use (diabetes 15.4%, no diabetes 2.1%; *p* <  0.001), use of cigarettes (diabetes 26.9%, no diabetes 4.3%; *p* <  0.001) and other drugs (diabetes 28.9%, no diabetes 4.0%; *p* <  0.001), cleanse/detox (diabetes 30.8%, no diabetes 10.5%; *p* <  0.001) and extreme weight loss diets (diabetes 13.5%, no diabetes 4.7%; *p* <  0.003) were higher in those reporting a diagnosis of diabetes. In addition, 17% of those with diabetes reported frequent insulin restriction (≥ once per week), and insulin restriction was associated with more frequent disordered eating behaviours.

**Conclusion:**

There was a high rate of disordered eating behaviours in adolescents with diabetes compared to their peers without diabetes. The findings of this study may have the potential to inform future health promotion, prevention, and early intervention approaches for those with comorbid diabetes and disordered eating behaviours. Future longitudinal studies are required to evaluate disordered eating behaviours in those with diabetes over time in community-based samples.

## Plain English summary

People with diabetes are at increased risk of developing disordered eating behaviours compared to people without diabetes. However, studies in the general community are needed to better understand disordered eating in adolescents with diabetes. We conducted an online survey in a large group of adolescents. This study found that several disordered eating behaviours were more common in adolescents with diabetes, including self-induced vomiting, laxative use, use of cigarettes and other illicit drugs, detox and extreme weight loss diets. This study may help to inform future treatments and prevention of disordered eating behaviours in adolescents with diabetes.

## Introduction

Eating disorders are complex mental health disorders and have one of the highest mortality and suicide rates of any mental illness [1, 2]. Meta-analytical data has shown that the prevalence of disordered eating behaviours are increasing in the general population [3–5] and a recent study found the point prevalence of any clinical or subclinical eating disorder to be 22% in Australian adolescents [6]. Another common health problem in adolescence is diabetes, predominantly Type 1 Diabetes (T1D) [7]. T1D is an auto-immune condition where the pancreas does not produce insulin. It is one of the most common chronic conditions in childhood and management requires careful monitoring of dietary intake and insulin use. In Type 2 Diabetes (T2D), the body becomes resistant to insulin or the pancreas does not produce adequate insulin. T2D is linked to modifiable lifestyle factors such as diet and exercise and is more common in adulthood; however, it is increasingly occurring in children and adolescents. Although there is no nationally representative data as to the proportion of adolescents affected by T1D in Australia, it is estimated that approximately 6400 Australian children and adolescents aged up to 14 years had T1D in 2016 [8]. While T2D is increasing in adolescence, it represents a far smaller proportion of adolescent diabetes, with an estimated prevalence of 0.01% in those aged 10–14 years and 0.04% in Australians aged 15–19 years [9].

People with diabetes have been shown to be an at-risk group for the development of disordered eating behaviours and consequently eating disorders compared to their non-diabetic peers [10]. Managing diabetes during adolescence can be challenging due to developmental and hormonal changes associated with this life stage [10]. This age also coincides with the peak incidence of eating disorders [11]. In a meta-analysis of 13 studies, 7% of adolescents with T1D were classified as having a diagnosable eating disorder, compared to 2.8% of adolescents without diabetes. Disordered eating behaviours such as self-induced vomiting, excessive exercise and laxative use have also been shown to be significantly more frequent among adolescents with T1D compared to those without diabetes [12]. While the cause of disordered eating in people with T1D is unclear, it may be due to several factors including the greater emphasis placed on monitoring dietary intake and maintaining a healthy weight, as well as insulin-related weight gain and resultant body dissatisfaction [12]. In T1D in particular, intentional insulin restriction is a unique disordered eating behaviour and method for rapid weight loss and caloric purging without the need for severe food restriction. While disordered eating patterns, particularly binge eating, have been identified in people with T2D [13], the majority of scientific literature has focused predominantly on binge eating behaviour in adults. Disordered eating behaviour including insulin restriction in people with diabetes is particularly concerning as it is associated with short-term physical complications including deteriorating glycaemic control and diabetic ketoacidosis, as well as long-term complications such as retinopathy, neuropathy, and premature death [14]. In addition, disordered eating behaviours are associated with impaired psycho-social functioning [15]. Given the ubiquity of disordered eating in diabetes and severity of associated complications, there is a need for a better understanding of this phenomenon to inform future intervention strategies, particularly during adolescence which is a developmental period of significant biological and social changes.

To date, the majority of studies investigating associations between diabetes and eating disorders have been conducted in relatively small samples drawn from clinical diabetes settings or registries [16], which may affect representativeness of the samples and generalisability to the broader population. Findings have also been mixed with regards to the impact of disordered eating on metabolic status [17] and very few studies have investigated community-based and population samples [18, 19]. Moreover, the majority of research has not assessed a broad spectrum of disordered eating behaviours [20], rather focusing on single aspects such as narrowly defined eating disorder diagnoses. Evaluating a range of disordered eating behaviours is needed to better characterise problematic eating patterns in this population, given the transdiagnostic nature of most eating disorder behaviours. Further, these behaviours may occur prior to eating disorder onset, and thus evaluating disordered eating in adolescents with T1D is important if we are to improve current treatment approaches, as well as screening and early intervention models to reduce the likelihood of progression to a diagnosable eating disorder. Studies in the general population, as opposed to clinical settings, are particularly timely and important to inform future community-based prevention and health promotion approaches that include vulnerable subgroups, such as those individuals with diabetes. Finally, community-based samples are required to fully understand the scope of the disordered eating among adolescents with diabetes in comparison to the non-diabetic population. Barriers to such an endeavour to date include the feasibility of recruiting large enough samples to allow detection of a sufficient group of participants with diabetes to facilitate meaningful analysis. The large, community-based sample in the present study provides a unique opportunity to undertake such an investigation.

The aim of this study was to evaluate a range of disordered eating behaviours in adolescents reporting a diagnosis of diabetes compared to their non-diabetic peers in a large community-based population sample of Australian adolescents, as well as to evaluate disordered eating behaviours according to insulin restriction in adolescents reporting diabetes. It was hypothesised that eating disorder behaviours would be higher in adolescents reporting diabetes compared to those without, and that insulin restriction would be associated with greater disordered eating behaviours.

## Methods

This is a secondary data analysis as part of the first wave of the EveryBODY study, a longitudinal investigation of eating disorders and body image concerns among Australian adolescents. Full details of the study are published elsewhere [6, 21]. Briefly, school principals and welfare staff of 50 secondary schools in the Hunter region, NSW, Australia, were contacted for participation. In an effort to improve ethnic diversity and representativeness, seven Sydney schools were subsequently invited to participate in the study. A final sample of thirteen schools participated in the study (*n* = 12 Hunter region, *n* = 1 Sydney). Government schools accounted for 67% of participating schools, followed by 33% Independent schools. Total enrolments across participating schools ranged from 514 to 1305 students, with approximately 70% of enrolled students at each school participating in the study. The school Index of Socio-Educational Advantage (ISCEA) of participating schools was close to the general population mean, however, there was less variation in socioeconomic status than the general population [21].

All students from participating school year groups were invited to participate in the online survey, which was completed at school under the supervision of teachers. Information regarding the study was provided to both parents and students in advance of testing. A passive parental consent procedure was used, whereby consent was assumed if parents did not actively opt their child out of the study. Students were required to provide online assent for participation on the day of the survey. At the end of the survey, students were provided with handouts, which contained information regarding resources and referral pathways for disordered eating and general mental health. All students who agreed were entered into a prize draw to win one of ten $100 gift cards. Ethics approval was received from the Macquarie University ethics committee and the New South Wales Department of Education.

### Measures

The online survey was comprised of self-reported items including demographics, self-reported height and weight, eating disorder behaviours, appearance-related scales, quality of life, mental health outcomes, social media activity, bullying and sexual and gender identities. The survey was pilot tested prior to its release to ensure the survey language was appropriate and could be completed within a 50-min class. As part of the current analysis, only variables relating to reported presence of diabetes, demographics, weight status, and disordered eating behaviours were included.

#### Demographics

Demographic variables were assessed including sex, age, country of birth, and postal code. Postal code was used to estimate participants area-level socioeconomic status (SES) using the Socioeconomic Index for Areas (SEIFA) Index of Relative Socioeconomic Advantage and Disadvantage (IRSAD), which classifies postcodes into deciles from 1 (most disadvantaged/least advantaged) to 10 (least disadvantaged/most advantaged) [22].

#### Self-reported diabetes diagnosis

Due to the community-based nature of the survey, as well as the age and comprehension levels of the adolescent sample recruited, a single dichotomous question was used to assess the presence of diabetes: “Have you ever been told by a doctor that you have diabetes?”. The question did not differentiate between T1D and T2D. Presence of diabetes (response options: yes/no) was used to categorise participants for analysis.

#### Anthropometrics

Participants were asked to self-report height (in centimetres) and weight (in kilograms), which was used to calculate body mass index (BMI) centile, adjusted for child age and gender. Online self-reported height and weight has previously been found to be valid for both adolescents and young adults [23, 24].

#### Eating disorders examination questionnaire

The Eating Disorders Examination Questionnaire (EDE-Q) was used to assess weight/shape concerns and disordered eating behaviours. The EDE-Q is a 28-item tool that assesses eating disorder pathology over the previous 28-days. The questions are scored using a 7-point Likert scale, with higher scores indicating greater eating disorder pathology. For the current study, only the weight and shape concern subscales (combined) and behavioural items were included. The weight and shape concern subscales include 12 items in total, which are averaged such that scores range from 0 to 6, with higher scores indicating more severe weight/shape concerns. This combined weight/shape concern scale has previously been validated in Australian adolescents [25]. Disordered eating behaviours for the purposes of weight or shape control that were assessed as part of the EDE-Q included objective binge eating, subjective binge eating, self-induced vomiting, use of laxatives, and driven exercise. Respondents are asked to indicate the frequency of these behaviours over the past 28 days using a free response format.

#### Other and atypical weight control Behaviour’s including insulin omission

Several additional behavioural frequency items were developed by the research team to assess other behaviours used for weight control purposes including: fasting for eight hours or more, cigarette smoking, other drug use, detoxes or cleanses, and strict weight loss diet. In addition, insulin restriction was assessed for those with diabetes using the following question: “*Over the past 4 weeks (28 days) how many times have you used less insulin (if you are diabetic) than you should have as a means of controlling your shape or weight?*”. Disordered eating behaviours were reported as frequency over the past 28 days. Frequency of at least once per week (i.e. 4 occasions per 28 days) was interpreted as frequent insulin restriction for the current study in line with the frequencies used for other behavioural symptoms in bulimia nervosa and binge eating disorder [26].

### Data analysis

A dedicated data set was derived of complete data for the main study variables in this study. Thus, from a total of 5191 participants who completed the survey, 337 respondents were excluded due to missing data on the variables of interest, resulting in a final sample of 4854 students for the current analyses. Participant characteristics were analysed descriptively, with data presented as frequencies for categorical data, mean (SD) for parametric data, and medians (IQR) for non-parametric data. To generate clinically meaningful cut-offs, disordered eating behaviours were transformed into a dichotomous variable. Consistent with previous research [6, 15, 25], the following cut-offs were used to categorise variables: *Any occurrence (*≥*1 time in past 28 days):* cigarette smoking, other drugs, detox/cleanse; *Weekly occurrence (*≥*4 times in past 28 days):* fasting, objective binge eating, subjective binge eating, self-induced vomiting, laxative use, insulin omission; *Greater than 3 days per week (*≥*13 times in past 28 days):* strict weight loss diet; *Five days per week or greater (*≥*20 times in past 28 days):* driven exercise.

For demographic comparisons between adolescents with and without diabetes, *t*-tests were used for continuous variables, while chi-squared tests were used for categorical variables. Univariate logistic regression models adjusted for age, sex and BMI centile were used to calculate odds ratios for disordered eating behaviours according to presence of diabetes. Data were grouped into younger (11–14 years) and older (15–19 years) adolescents to assess if there were any associations between disordered eating behaviours and age. Due to the low numbers of participants with diabetes who reported restricting insulin, Mann Whitney-U tests for continuous data and Fishers Exact tests for categorical data were used to compare these participants to those with diabetes who did not report restricting insulin.

## Results

Participant characteristics are presented in Table 1. The mean age of participants was 14.4 (±1.6) years (range 11–19 years) and 47% were boys. Little’s MCAR test demonstrated data were not missing at random (*p* <  0.001). Compared to the current study sample, survey non-completers had a higher proportion of males (completers 47%, non-completers 59%; *p* <  0.001), were older (completers 14.4 years, non-completers 15.2 years; *p* <  0.001) and had a higher BMI centile (completers 54.1, non-completers 58.8; *p* = 0.03). The majority of participants were in Grade 7 (*n* = 1069), followed by Grade 10 (*n* = 1047), Grade 8 (*n* = 1011), Grade 9 (*n* = 915), Grade 11 (*n* = 543) and Grade 12 (*n* = 269). The mean BMI centile was 54.1 (± 30.9) with 65% classified as normal weight. Participants were from a range of socioeconomic backgrounds with a mean SEIFA decile of 5 (range 1–10). A total of 52 (1.1%) adolescents self-reported receiving a diagnosis of diabetes from a doctor. The diabetes group had a higher proportion of participants within the obese BMI category (*p* <  0.001).

**Table 1:** Participant characteristics according to presence of diabetes

	Total sample (*n* = 4854)	Diabetes (*n* = 52)	No diabetes (*n* = 4802)	*p*
	*Mean (SD)*			
Age *mean (SD)*	14.4 (1.6)	14.5 *(*1.9)	14.4 (1.6)	0.55
BMI percentile *mean (SD)*	54.1 *(*30.9)	61.7 *(*27.7)	54.0 *(*30.9)	0.14
SEIFA *mean (SD)*	985.0 *(*42.0)	977.9 *(*41.3)	985.1 *(*42.0)	0.24
SEIFA decile *mean (SD)*	5.3 *(*1.9)	5.6 *(*1.6)	5.3 *(*1.9)	0.30
	***% (n)***			
Sex [boys]	47.0 (2282)	59.6 (31)	46.9 (2251)	0.07
Weight category				
Underweight	7.5 (366)	3.9 (2)	7.6 (364)	
Normal Weight	64.8 (3144)	51.9 (27)	64.9 (3117)	
Overweight	12.6 (612)	7.7 (4)	12.7 (608)	
Obese	15.1 (732)	36.5 (19)	14.9 (713)	**<.001**
Country of birth				
Australian	89.6 (4347)	84.6 (44)	89.6 (4303)	
European	2.1 (102)	1.9 (1)	2.1 (101)	
Asian	5.5 (267)	5.8 (3)	5.5 (264)	
Other	2.8 (138)	7.7 (4)	2.8 (134)	0.26

Notes: *BMI* body mass index, *SEIFA* Socioeconomic Index for Areas

### Disordered eating behaviours

Disordered eating behaviours according to diabetes status are presented in Table 2. Those with diabetes reported higher frequency of disordered eating behaviours including self-induced vomiting, laxative use, use of cigarettes and other drugs for weight or shape control, and cleanse/detox and extreme weight loss diets, compared to those with no diabetes. Adolescents with diabetes had between 2.7 and 6.3 greater odds of reporting several disordered eating behaviours, especially those associated with weight control. The behaviours that were most strongly associated with diabetes, controlling for demographic differences, were the use of cigarettes, laxatives and other drugs for weight control purposes. Weight and shape concerns, fasting, driven exercise and binge eating were also higher in participants reporting diabetes, however this did not reach statistical significance. No significant associations were identified between disordered eating behaviours and age group.

**Table 2:** Frequency and odds of eating disorder behaviours, weight and shape concern according to reported presence of diabetes

	Total sample (*N* = 4854)	Diabetes (*n* = 52)	No diabetes (*n* = 4802)	*x*^2^ (*p*) ^a^	Odds ratio (95% CI) ^b^
	*% (n)*				
EDE Q Disordered eating behaviours					
Fasting	10.5 (511)	9.6 (5)	10.5 (506)	0.1 (0.83)	0.2 (0.03, 1.7)
Objective binge eating	17.4 (844)	25.0 (13)	17.3 (831)	2.1 (0.15)	1.1 (0.5, 2.5)
Subjective binge eating	17.2 (834)	21.2 (11)	17.1 (823)	0.1 (0.45)	0.4 (0.1, 1.4)
Self-induced vomiting	3.5 (170)	19.2 (10)	3.3 (160)	**38.5 (< 0.001)**	3.9 (1.3, 11.3)
Laxative use	2.2 (108)	15.4 (8)	2.1 (100)	**41.8 (< 0.001)**	4.7 (1.4, 15.7)
Driven exercise	5.5 (268)	3.9 (2)	5.5 (266)	0.3 (0.60)	1.0
Cigarette use	4.6 (222)	26.9 (14)	4.3 (208)	**60.2 (< 0.001)**	4.8 (1.9, 11.7)
Illicit drug use	4.2 (206)	28.9 (15)	4.0 (191)	**78.3 (< 0.001)**	6.3 (2.7, 14.7)
Detox or cleanse diet	10.7 (519)	30.8 (16)	10.5 (503)	**22.2 (< 0.001)**	2.7 (1.2, 6.2)
Extreme weight loss diet	4.8 (232)	13.5 (7)	4.7 (225)	**8.7 (0.003)**	1.8 (0.5, 6.0)
	*Mean (SD)*				
EDEQ Shape concern *mean (SD)*	1.6 (1.8)	2.1 (2.1)	1.6 (1.8)	0.08 ^c^	
EDEQ Weight concern *mean (SD)*	1.5 (1.8)	1.9 (2.0)	1.5 (1.8)	0.14 ^c^	

Notes: ^a^ Chi 2 test; ^b^ Univariate odds ratios adjusted for age, sex and BMI centile; ^c^ t test *p* value; *EDEQ* Eating Disorders Examination Questionnaire

### Insulin restriction

Nine participants (17%) reporting diabetes reported regular insulin restriction for the purpose of weight loss (frequency of ≥1 per week). There were no significant differences between groups for age or BMI centile. Disordered eating behaviours were significantly higher in those reporting insulin restriction compared to those not restricting insulin including (all Fishers Exact) fasting (33.3% vs 4.7%, respectively; *p* = 0.03), objective binge eating (77.8% vs 14.0%, respectively; *p* < 0.001), subjective binge eating (77.8% vs 9.3%, respectively; *p* < 0.001), self-induced vomiting (66.7% vs 9.3%, respectively; *p* = 0.001), laxative use (77.8% vs 2.3%, respectively; *p* < 0.001), driven exercise (22.2% vs 0%, respectively; *p* = 0.03), cigarette use (77.8% vs 16.3%, respectively; *p* = 0.001), illicit drug use (77.8% vs 18.6%, respectively; *p* = 0.001), detox or cleanse diet (100.0% vs 16.3%, respectively; *p* < .0.001), extreme weight loss diet (44.4% vs 7.0%, respectively; *p* = 0.01). Weight concern [median (IQR); 3.6 (1.4–5) vs 0.6 (0–3.2), respectively; *p* = 0.03] and shape concern [median (IQR); 3 (2–5.5) vs 1.5 (0–3.8), respectively; *p* = 0.03] were also higher in those reporting insulin restriction compared to those not restricting.

## Discussion

This study aimed to explore disordered eating behaviours according to reported diagnosis of diabetes and insulin restriction in a general community-based population sample of adolescents. To the authors’ knowledge, this study has assessed the broadest spectrum of disordered eating behaviours in the context of diabetes to date. Several disordered eating behaviours were greater in those with a self-reported diagnosis of diabetes, including self-induced vomiting, laxative use, cleanse or detox diets, extreme weight loss diets as well as cigarette and drug use for the purposes of weight or shape control. In addition, frequent insulin restriction was found to be common in those who reported diabetes and was associated with even greater rates of disordered eating.

The behaviours that were particularly common among youth with diabetes compared to their peers included purging behaviours (i.e., self-induced vomiting and laxative use) and atypical substance-related weight control behaviours (use of detoxes, cigarettes and other drugs). The rates of self-induced vomiting (19%) and laxative use (15%) among adolescents with diabetes in this sample were higher than previous studies of adolescents with diabetes in clinical settings (4 and 1%, respectively [27, 28];). This may be attributable to the community-based, anonymous nature of the current survey compared to recruitment from diabetes clinics in previous studies. Further, while purging behaviours have frequently been assessed in people with diabetes, the frequency of purging is often unreported, with studies instead relying on global measures of eating disorder psychopathology [16]. Detoxes and extreme weight loss dieting were frequently reported in adolescents with diabetes in this study. This type of behaviour may be triggered among youth diagnosed with diabetes who are taught to closely monitor their dietary intake and weight during a time where dieting is a known casual risk factor for eating disorder onset among non-diabetic youth [29]. The use of extreme weight control strategies is especially concerning among adolescents with diabetes as this may affect their overall blood glucose management.

The use of cigarettes [30] and other illicit drugs [31–33] for the purposes of weight and shape control has been previously documented in the general population and those with clinical and subclinical eating disorders, however, the frequency of these behaviours among people with diabetes has been understudied. In the current study, we found that adolescents with diabetes were almost 5-times more likely to report cigarette use and more than 6-times more likely to report illicit drug use for weight control purposes compared to their non-diabetic peers. Cigarette smoking in people with diabetes poses increased health risks including eye and nerve problems, hyperglycaemia and poor glycaemic control, while use of illicit drugs may be particularly dangerous for youth with diabetes if they consequently reduce their dietary intake or forget to take insulin, which has the potential to lead to poor glycaemic control. Given the unique medical implications of these disordered eating behaviours for people with diabetes, these findings indicate that it is important to assess a range of disordered eating behaviours in clinical diabetes settings and tailor interventions to be specific to those with diabetes. Adolescents who reported diabetes were not more likely to engage in frequent binge eating behaviour, in both unadjusted and adjusted models that included body mass index. This was contrary to expectations, given the strict dietary control required in diabetes, which is also widely regarded as a risk factor for binge eating [34]. Indeed, previous research has found that among adolescents who intentionally overdose on insulin, the primary reason for this was to allow themselves to binge eat [35]. However, this finding may also be because binge eating is more commonly found in adults, including those with T2D [36], and less prevalent in adolescents. More research is required to determine whether adolescent T1D is associated with greater risk of binge eating or not.

Within the diabetes group, 17% reported insulin restriction at least once per week in order to control weight. Insulin restriction for the purpose of weight or shape control has been widely reported in previous literature ranging from 4 to 58% in people with T1D [37]. Insulin restriction in the current study identified a vulnerable subgroup displaying very high rates of eating disorder behaviours, which is similar to previous studies in male and female adults [38]. Disordered eating behaviours have also been shown to be associated with insulin restriction in adolescents [18, 39]. Furthermore, weight and shape concern were higher in those that reported restricting insulin. The use of insulin can result in weight gain, which may contribute to body dissatisfaction, a leading risk factor for the development of disordered eating [40]. However, due to the cross-sectional nature of the study, we are unable to determine causal pathways for the development of weight and shape concern and disordered eating behaviours among youth with diabetes. Consistent with existing guidelines [10], paediatric diabetes clinics should routinely screen for disordered eating behaviours, including insulin omission. Strategies to address insulin omission including psychoeducation should be employed to minimise likelihood of progression to a diagnosable eating disorder. This is important given that identification and referral to timely and appropriate care provision may reduce subsequent complications and improve treatment outcomes [41].

The findings of this study may have the potential to inform future health promotion, prevention, and early intervention approaches. Given the differences in disordered eating behaviours according to diabetes status, selective programs for those with diabetes may be preferable, given that there are unique disordered eating behaviours in this group. Alternatively, existing health promotion and universal prevention programs may consider adding diabetes-specific information, such as insulin restriction, given the serious medical complications associated with this behaviour. In addition, education for parents and health care professionals may also be useful to assist in identifying signs of disordered eating behaviours and referral to appropriate treatment pathways.

Strengths of this study include the recruitment of a large, general community-based population sample of adolescents across a broad range of demographic characteristics, with many previous studies recruiting convenience samples of people with diabetes from clinical settings. However, this study has some limitations to acknowledge. The self-reported diabetes question was worded to be age appropriate, but it did not differentiate between T1D and T2D. Given the age of the sample recruited, it can be assumed that the majority of participants had T1D as this is the predominant form of diabetes in this age group. However, the group with diabetes also had a higher BMI, which is associated with increased risk for T2D. Due to the different biological mechanisms, morbidity and management strategies in T1D compared to T2D, future studies should ensure that the type of diabetes is specific in the question, to allow for analyses according to diagnosis. In addition, as this was a secondary analysis as part of a larger study, an eating disorders assessment tool specific to diabetes was not used. Previous research has suggested that prevalence estimates of diagnosable eating disorders should be interpreted with caution in people with diabetes [17], as many eating disorder assessment tools for the general population include questions that pathologise behaviours that are important for management of T1D (for example the focus on food intake) and may inflate the prevalence of disordered eating. To address this limitation, this study specifically explored specific eating disorder behaviours rather than eating disorder diagnosis. The cross-sectional nature of the study precludes inferences about cause and effect with respect to the emergence of diabetes and eating disorders. Future longitudinal community-based studies are required and studies that investigate the impact of disordered eating on metabolic and other indices of physical as well as mental health status in young people with diabetes. The small number of participants in the diabetes group, and particularly the insulin restriction subgroup, is a limitation of the study. However, differences between the two subgroups were nevertheless observed suggesting there was sufficient power in the analyses. Finally, assessment of insulin over-dosing in addition to insulin restriction is recommended in future studies of this kind, given the reported association with binge eating behaviour.

## Conclusions

This study found that disordered eating behaviours including self-induced vomiting, laxative use, cleanse or detox diets, extreme weight loss diets as well as cigarette and drug use were higher in adolescents reporting a diagnosis of diabetes in a community-based population sample of Australian adolescents, a particularly vulnerable subgroup due to the rapid biological and social changes during this stage of development. In addition, frequent insulin restriction was found to be common in those who reported a diagnosis of diabetes and was associated with very high rates of disordered eating behaviours and weight and shape concern. Future longitudinal studies are required to evaluate disordered eating behaviours in those with diabetes over time in community-based samples.

## Data Availability

Readers are encouraged to contact the corresponding author for data requests.

## Abbreviations

BMI: Body mass index
EDE-Q: Eating Disorders Examination Questionnaire
IRSAD: Index of Relative Socioeconomic Advantage and Disadvantage
SD: Standard deviation
SEIFA: Socioeconomic index for areas
SES: Socioeconomic status
T1D: Type 1 diabetes
T2D: Type 2 diabetes
